# Tokyo DoManNaka: Learning opportunities of clinical diagnostic reasoning from expert physicians

**DOI:** 10.1002/jgf2.334

**Published:** 2020-05-17

**Authors:** Ayaka Takahara, Kiyoshi Shikino, Takashi Watari, Osamu Hirashima, Satoshi Watanuki, Masatomi Ikusaka

**Affiliations:** ^1^ Chiba University School of Medicine Chiba Japan; ^2^ Department of General Medicine Chiba University Hospital Chiba Japan; ^3^ Postgraduate Clinical Training Center Shimane University Hospital Shimane Japan; ^4^ Department of General Medicine Naze Tokushukai Hospital Kagoshima Japan; ^5^ Division of Emergency and General Medicine Tokyo Metropolitan Tama Medical Center Tokyo Japan

To the editor

In Japan, the specialty of general medicine was started in April 2018 as the 19th basic area in the medical specialty board system. However, the number of general medicine residents was only 184 (2.2% of the total) in 2018 and 179 (2.1% of the total) in 2019.^1^ Clinical diagnostic reasoning is cited as the factor that effects most strongly and positively for medical students to consider a career as a general medicine physician.^2^ In addition, guidance from expert physicians also works positively for career choice.^3^ Based on this evidence, a student study group to learn clinical diagnostic reasoning from expert physicians called "Tokyo DoManNaka", which means "right in the middle of Tokyo", was launched in March 2019.

Tokyo DoManNaka aims at providing students with practical clinical reasoning skills from the viewpoint of expert physicians. The conference is held twice a year, and more than 100 medical students and early residents from across Japan participate in the course. Each time, 4‐5 expert physicians in the field of general medicine are invited as lecturers. Medical students can learn about clinical diagnostic reasoning directly from the expert physician, who is a role model for generalists, and can deepen their interest in the field of general medicine.

Tokyo DoManNaka is an autonomous organization based on cooperation between students and general practice instructors. In terms of publicity, we co‐organize Tokyo DoManNaka along with other student organizations and gather stakeholder student staff from each university to inform them about this organization. We also use SNS, such as Facebook to attract more participants. There is no charge for participation, and we are trying to make it easy for students to participate. The main staff assesses the needs of the participants and recruits expert physicians who have expertise in the subject.

In the first seminar, we conducted the training in five sections: 1) a session to learn the importance of Bayesian theory in clinical reasoning, 2) a workshop to think about the causes of diagnostic errors and what can be done to reduce them, 3) a case study conference to learn the process of analytical diagnostic reasoning, 4) a session to learn physical examinations useful in clinical practice, and 5) a case simulation using simulated patients.

A questionnaire was conducted using a 5‐point Likert scale (from 1: strongly disagree to 5: strongly agree) concerning whether the learning objectives have been achieved. The mean score was rated as 4.6 on average, and 61 (95.3%) of the 64 participants gave a score of 4 or 5.

The appeal of Tokyo DoManNaka is that medical students can learn clinical diagnostic reasoning from expert physicians in the field of general medical, which enables them to consider the diagnosis of a case and practice it on their own initiative. Educational seminars on clinical diagnostic reasoning have been effective for postgraduate physicians in Japan.^4^ Along the same lines, Tokyo DoManNaka hopes to teach medical students about the fun and depth of clinical reasoning and how to think about comprehensive medical care.

## CONFLICT OF INTEREST

The authors have stated explicitly that there are no conflicts of interest in connection with this article.
